# Comparative analysis reveals regulatory motifs at the *ainS*/*ainR* pheromone-signaling locus of *Vibrio fischeri*

**DOI:** 10.1038/s41598-017-11967-7

**Published:** 2017-09-15

**Authors:** John H. Kimbrough, Eric V. Stabb

**Affiliations:** Department of Microbiology, University of Georgia, Athens, GA USA

## Abstract

*Vibrio fischeri* uses the AinS/AinR pheromone-signaling system to control bioluminescence and other symbiotic colonization factors. The Ain system is thought to initiate cell-cell signaling at moderate cell densities and to prime the LuxI/LuxR signaling system. Here we compared and analyzed the *ain* locus from two *V*. *fischeri* strains and a *Vibrio salmonicida* strain to explore *ain* regulation. The *ainS* and *ainR* genes were predicted to constitute an operon, which we corroborated using RT-PCR. Comparisons between strains revealed a stark area of conservation across the *ainS*-*ainR* junction, including a large inverted repeat in *ainR*. We found that this inverted repeat *in cis* can affect accumulation of the AinS-generated pheromone *N-*octanoyl homoserine lactone, which may account for the previously unexplained low-signal phenotype of a ∆*ainR* mutant, although the mechanism behind this regulation remains elusive. We also extended the previous observation of a possible “*lux* box” LuxR binding site upstream of *ainS* by showing the conservation of this site as well as a second putative *lux* box. Using a plasmid-based reporter we found that LuxR can mediate repression of *ainS*, providing a negative feedback mechanism in the Ain/Lux signaling cascade. Our results provide new insights into the regulation, expression, and evolution of *ainSR*.

## Introduction

The light-organ symbiont *Vibrio fischeri* ES114 uses pheromone signaling (PS) to regulate behaviors essential for colonizing its host squid, *Euprymna scolopes* ^1–7^. One of these behaviors, bioluminescence, was among the first phenotypes discovered to be regulated by PS, and its examination over the last fifty years has been fundamental to understanding bacterial cell-cell communication^8^. Of *V*. *fischeri*’s three integrated PS systems, two acyl-homoserine lactone (AHL)-based systems are primarily responsible for regulating bioluminescence and other colonization factors, while the autoinducer-2 (AI-2) signal, which is conserved across many bacteria, only modestly influences these phenotypes under the conditions tested^2^.

The two AHL-based PS systems in *V*. *fischeri* that regulate bioluminescence and other symbiotic factors are comprised of the signal synthase/receptor pairs LuxI/LuxR and AinS/AinR^1,4,5^. LuxI and AinS produce AHL molecules that can diffuse through membranes and mediate cell-cell signaling once they reach stimulatory concentrations^9^. LuxI synthesizes the pheromone *N-*3-oxohexanoyl homoserine lactone (3OC6-HSL)^10,11^, which binds LuxR, promoting LuxR dimerization and association with a regulatory element upstream of *luxI* called the “*lux* box”^9,12^. AHL-LuxR complexes activate transcription of the *luxICDABEG* operon, which results in more LuxI, more 3OC6-HSL, and bioluminescence. LuxI and LuxR are well studied and are the archetype for similar PS systems throughout the Proteobacteria.

Although less well known, the structurally distinct AinS/AinR PS system also uses an AHL signal and plays key roles in luminescence induction and symbiotic competence^3^. AinS synthesizes the pheromone *N-*octanoyl homoserine lactone (C8-HSL), which is sensed by AinR^13,14^ but can also activate LuxR^15,16^. Information about local C8-HSL concentration is funneled by AinR into a core PS system conserved among the *Vibrionaceae*, converging with the AI-2 system, to affect bioluminescence through a regulatory cascade comprised of LuxU, LuxO, the sRNA Qrr, and the terminal regulator of the system, which is called LitR in *V*. *fischeri* ^17–20^. LitR activates LuxR expression and, as noted above, C8-HSL can activate LuxR directly, albeit more weakly than 3OC6-HSL. Thus, in more than one way the Ain system serves to activate *lux* expression and “prime” 3OC6-HSL-based signaling^3,15–17^. However, at certain AHL ratios, C8-HSL can actually inhibit 3OC6-HSL-based activation^15,21–23^.

In *V*. *fischeri* strain ES114, which was isolated from the light organ of *E*. *scolopes*, the Ain system is critical for induction of luminescence in broth culture and underlies regulation of symbiotic colonization factors. Given this role, understanding the control of *ainS/ainR* will provide important insight into gene regulation during establishment of the symbiosis. LitR activates the *ain* system, closing a positive feedback loop^2,13^, and CRP-cAMP was recently identified as an activator of both *ainS/ainR* and *luxI/luxR* ^24^, although the connections between CRP, cAMP, and carbon source are not entirely clear in *V*. *fischeri* ^25,26^. Given the complex regulation of *luxI* and *luxR* ^23,24,27–33^, we predict that other regulatory mechanisms of *ain* regulation await discovery. Indeed, studies have suggested that the presence of *ainR in cis* somehow affects AinS activity^13,18^ and that a feedback loop exists between LuxR and the *ain* system^13^.

We previously reported that both the *lux* and *ain* loci have evolved rapidly and diverged between *V*. *fischeri* strains more rapidly than most housekeeping genes^34^. Moreover, we found that comparison of the *luxIR* intergenic region between strains provided insight into conserved regulatory sequences^34^. In this study we similarly used bioinformatic comparisons and targeted experimentation to gain insight into expression of the *ain* locus.

## Results

### Comparative analysis of the ainS/ainR locus

**Figure 1:**
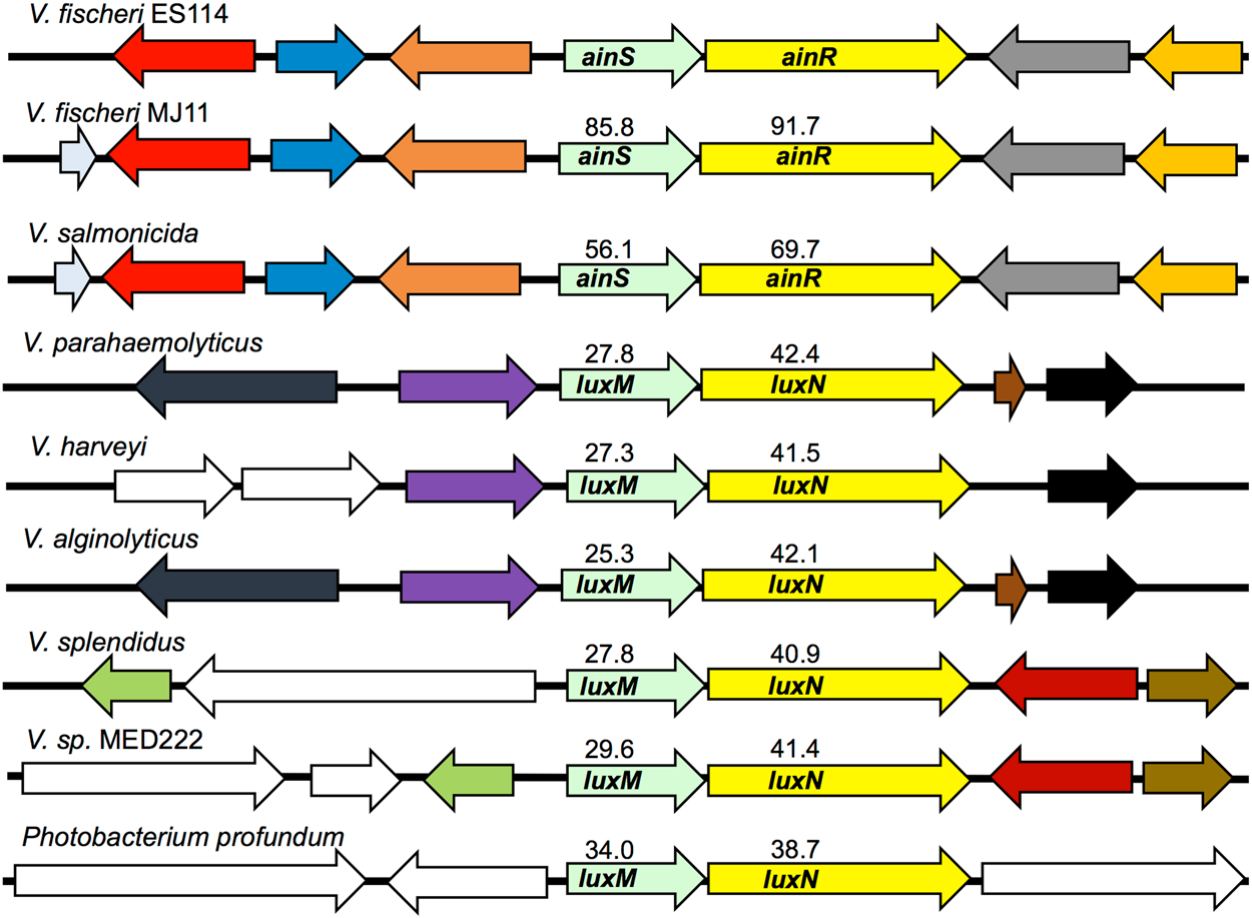
Homologs of *V*. *fischeri* AinS/AinR and synteny around the *ain* locus. Aligned sequences are from *V*. *fischeri* ES114 and MJ11, *V*. *salmonicidia* LF1238, *V*. *parahaemolyticus* RMID2210633, *V*. *harveyi* ATCC BAA-1116, *V*. *alginolyticus* 12G01, *V*. *splendidus* 12B01, *Vibrio* spp. MED222, and *Photobacterium profundum* SS9. Arrows of the same color share homology, white arrows have no homologs in the figure, and numbers indicate percent identity to AinS or AinR from *V*. *fischeri* ES114. The 10**-**kb region encompassing *V*. *fischeri* ES114 *ainS* is shown, and synteny was assessed using the SEED^44^ database.

Homologs of *V*. *fischeri* AinS and AinR include *Vibrio harveyi* LuxM and LuxN^35^, which were the first members of this type of PS system described, as well as other pairs of similar ORFs in *Vibrio salmonicida*, *Vibrio parahaemolyticus*, *Vibrio alginolyticus*, *Vibrio splendidus*, *Vibrio* sp. MED222, and *Photobacterium profundum* (Fig. 1). Unlike LuxI and LuxR homologs, signaling systems similar to AinS and AinR have not yet been identified outside the *Vibrionaceae*. Most of the loci shown in Fig. 1 lack synteny or useful DNA sequence conservation with *V*. *fischeri ainS*/*ainR*. However, we were able to effectively compare this locus in three strains; *V*. *fischeri* ES114^36,37^, which is a dimly luminescent strain characteristic of other isolates from *E*. *scolopes*, *V*. *fischeri* MJ11^38,39^, which is a bright isolate from the Japanese pinecone fish *Monocentris japonica*, and *Vibrio salmonicida* LFI1238^40^, which was isolated from a diseased cod. ES114 and MJ11 represent different clades of *V*. *fischeri* ^34,39^, whereas *V*. *salmonicda* is a closely related fish pathogen. Genome sequences are available for all three strains^37,39,40^.

**Figure 2:**
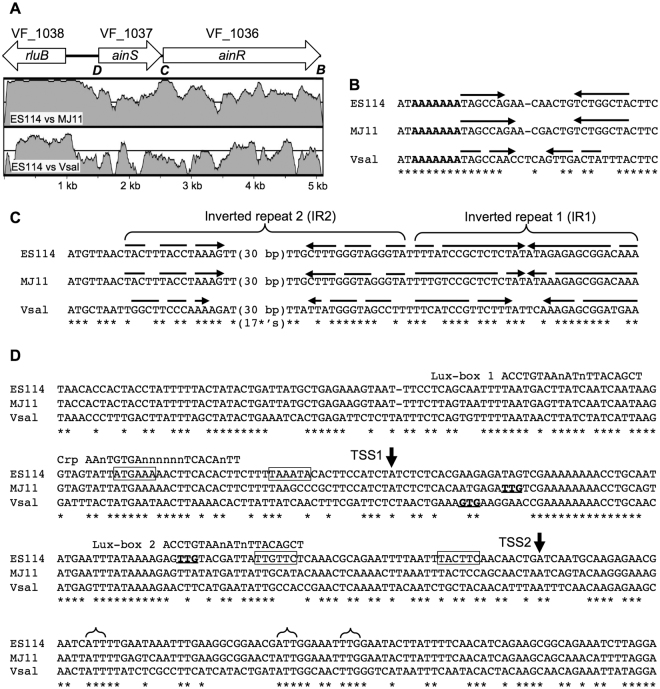
Comparison of the *ainSR* locus in three *V*. *fischeri* and *V*. *salmonicida* strains. The sequence between the stop codons of VF_1038 and VF_1035 in *V*. *fischeri* ES114 was compared to the orthologous regions from *V*. *fischeri* MJ11 and *V*. *salmonicida* LFI1238. In panel A, arrows show the arrangement of the three complete genes at this locus, which extends to the stop codon of VF_1035 on the right. Bold and italicized letters under the arrows indicate regions for which DNA sequence is shown in the corresponding panels. Homology between ES114 and MJ11 or *V*. *salmonicida* is shown for corresponding regions in shaded plots that range from fifty to one hundred percent identity within a 100-bp window as determined by VISTA^73^ with the LAGAN^74^ alignment function and default settings. The grey line denoted seventy-five percent identity. Panel B shows a conserved putative Rho-independent transcriptional terminator downstream of VF_1035 that was identified by ARNold^42^, with arrows indicating inverted repeat stems, with stem mismatches indicated by gaps on the arrows. A bold-lettered run of A’s indicates the canonical string of U’s (on the reverse strand transcript) following a stem loop structure in such terminators. Panel C shows sequences aligned from the start codon of *ainR*, with arrows indicating inverted repeats and mismatches indicated by gaps on the arrows. Panel D shows an alignment of sequences upstream of and within *ainS*. Two transcriptional start sites mapped in strain ES114 by 5′ RACE are indicated as TSS1 and TSS2, and putative −10 and −35 promoter elements associated with TSS1 and TSS2 are boxed. Start codons predicted by The SEED^44^ are indicated with bold and underlined letters. Brackets indicate alternative start codons conserved across all three strains. A CRP binding site^24^ and possible “*lux* box” LuxR-binding sequences are highlighted by alignment with the respective consensus binding sequence. In panels B–D, asterisks indicate bases conserved in all three strains.

As previously reported^34^, the *ainS* and *ainR* genes have diverged more between ES114 and MJ11 than have most other orthologs in these strains, including the well conserved *rluB* gene adjacent to *ainS* (Fig. 2A). This trend was also evident in a comparison of ES114 and *V*. *salmonicida* (Fig. 2A). In all three strains there is only an 11-bp gap between the stop codon of *ainS* and the start codon of *ainR*, and the DOOR operon-prediction database indicated that *ainS* and *ainR* are likely to be co-transcribed^41^. Consistent with that prediction, RT-PCR indicated that *ainS* and *ainR* sequences can be found on the same RNA, as evidenced by an appropriately sized RT-PCR product that was absent in a no-RT control or when mRNA from ∆*ainS* or ∆*ainR* mutants was used (see Supplementary Figure S1). Using ARNold^42^, we further identified a putative Rho-independent transcriptional terminator between *ainR* (ORF VF_1036) and the adjacent convergent ORF VF_1035 (Fig. 2B). Based on its sequence, this putative terminator appears uni-directional and more likely to terminate the VF_1035 transcript than the *ainSR* transcript.

Despite the relatively low homology between the *ainSR* loci in ES114 and MJ11, there is a short stretch of high sequence identity spanning the downstream end of *ainS* and the beginning of *ainR* (Fig. 2A and C). A striking feature within this portion of *ainR* is a nearly perfect 32-bp inverted repeat element designated IR1 (Fig. 2C). IR1 is also evident in *V*. *salmonicida*, although the sequence has diverged from that of the *V*. *fischeri* strains (Fig. 2C). RNA-folding predictions revealed another inverted repeat (designated IR2), although there are more mismatches between repeats and the gap between the repeats in IR2 is over 30 bp (Fig. 2C).

To help identify possible mechanisms of *ainSR* regulation, we mapped the *ainS* transcriptional start site(s) in ES114 using 5′-RACE and overlaid the results on an alignment of sequences upstream of *ainS*. After sequencing ten RACE clones, we found a nearly even distribution of two distinct 5′ transcript ends, which are shown in Fig. 2D labeled as TSS1 (four clones) or TSS2 (six clones). Boxed sequences upstream of TSS1 and TSS2 in Fig. 2D represent possible −10 and −35 promoter elements, based on reasonable matches to these elements and their spacing at *Escherichia coli* sigma-70 promoters^43^. Although this analysis must be viewed cautiously, there is enough conservation of key putative-promoter elements between the strains to suggest that TSS1 and TSS2 are not unique to ES114.

The translational start(s) for AinS was difficult to place definitively. Annotation by the SEED^44^ predicted non-canonical (non-ATG) start codons for *ainS* in each of the three strains, but the position of the predicted start is different in each strain (Fig. 2D). None of these predicted start sites is conserved between the three strains and none match the translational start suggested by Gilson *et al*. for AinS in strain MJ1^45^. Furthermore, each of the predicted translational start sites is upstream of TSS2 and would not be present on transcripts that initiate at this position (Fig. 2D). Other recent studies (e.g., Nakahigashi *et al*.^46^) have underscored the potential for mistakes with automated annotation of translational starts as well as the prevalence of multiple start sites for particular genes. Accordingly, it seemed worth re-examining possible translational starts for AinS. It seems likely that the canonical *E*. *coli* ribosome binding site (RBS) sequence serves the same function in *V*. *fischeri* and *V*. *salmonicida*, because all three species are identical across the critical 3′ end of the 16S rRNA that forms complementary base pairing with the Shine-Dalgarno sequence on transcripts (data not shown). Among non-ATG start codons, GTG and TTG are the most common and are often the only possibilities considered by automated annotation programs; however, CTG and ATT start codons have been documented as well^46–48^. We identified two ATT codons and one TTG that are conserved across all three strains (Fig. 2D), and a putative ribosome-binding site (RBS) is well conserved across all three strains for the first (furthest upstream) ATT. Moreover, the putative RBS sequences near these potential starts appear to be as good or better matches than those for previously annotated starts. These putative non-ATG start codons also occur downstream of TSS2 but before AinS residues begin to align with similarity to AinS orthologs found in other members of the *Vibrionaceae*.

Previous examination of the *ainS* promoter region identified a CRP binding site^24^ and a putative *lux* box just upstream of the CRP site^45^, each of which are reasonably well conserved across the three strains (Fig. 2D). We also identified a second potential *lux* box overlapping the putative −35 promoter element associated with TSS2 (Fig. 2D). No other potential regulatory sequences were immediately apparent, although implications of our findings for regulation by CRP and LitR are discussed below. We focused experimentally on the putative *lux* box elements and IR1 for their potential role in *ainSR* control.

### *luxR*-mediated repression of *ainSR*

**Figure 3:**
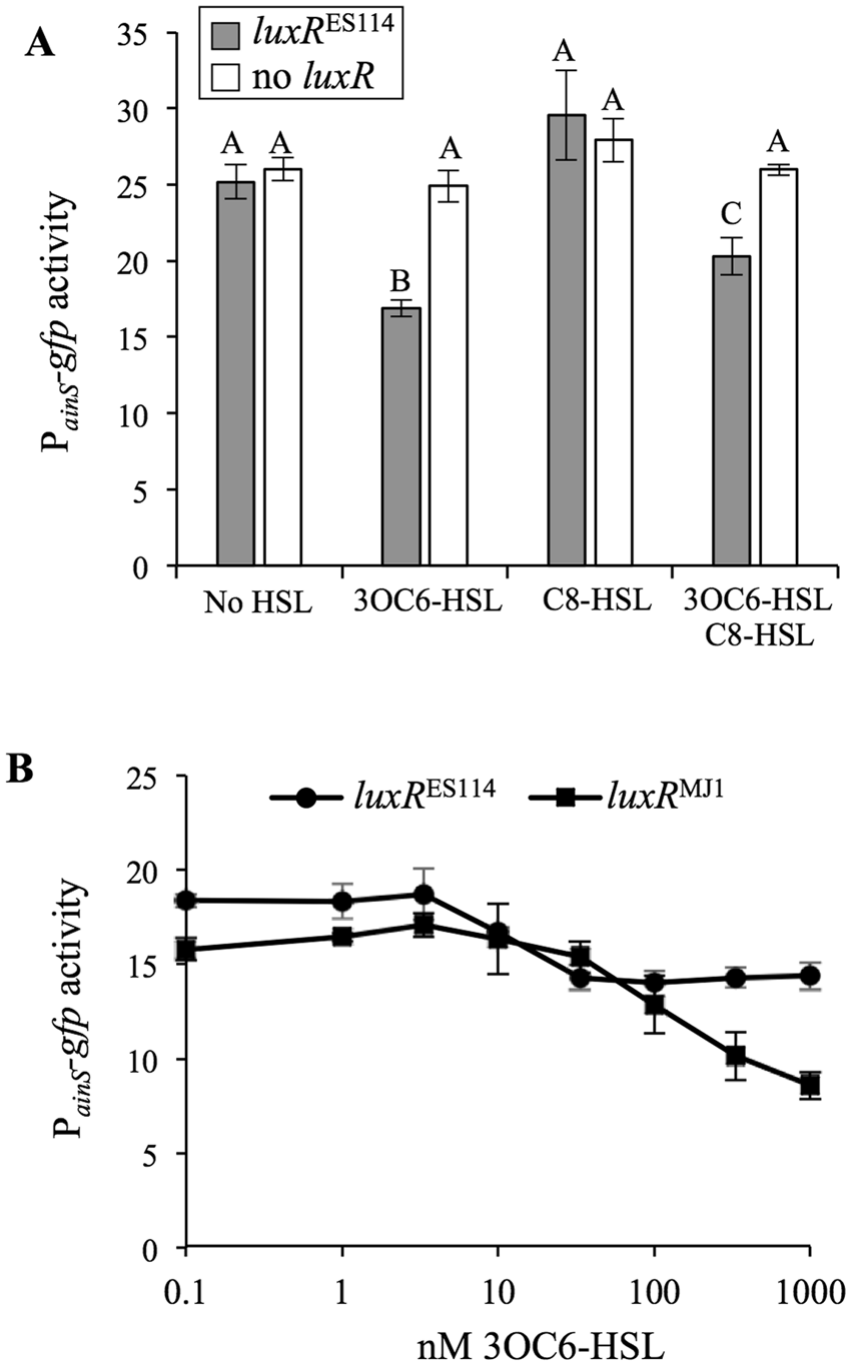
LuxR-3OC6-HSL represses a P_*ainS*_ *-gfp* reporter. Both panels report fluorescence of cells harboring P_*ainS*_ *-gfp* reporter pHK12 grown in SWTO to an OD_595_ of ~2.5. Strains harboring the reporter lack AHL synthases (∆*luxI* ∆*ainS*) and feedback regulation of *luxR* (*litR*::*ermR* P_con_ *-luxR*), and either express *luxR* ^ES114^ (JHK045), *luxR* ^MJ1^ (JHK099), or no *luxR* (JHK046). Panel A: Strains with *luxR* ^ES114^ (grey bars) or no luxR (white bars) harboring pHK12 and supplemented with 500 nM 3OC6-HSL and/or C8-HSL. Treatments marked with different letters are significantly different (*P* < 0.05) as determined by one-way ANOVA. Panel B: Strains with *luxR* ^ES114^ or *luxR* ^MJ1^, carrying pHK12, in media with varied 3OC6-HSL concentrations. In both panels, bars indicate standard error (*n* = 3).

We sought to clarify the role, if any, of LuxR in regulating *ainSR*. Others had identified one of the putative *lux* boxes upstream of *ainS* ^45^, and we previously reported a LuxR-dependent decrease in P_*qrr*_-*lacZ* reporter activity, which might be due to LuxR repression of the *ainSR* promoter^13^. On the other hand, *ainS* was not identified as part of the LuxR regulon^49,50^, although as discussed below those studies are arguably not definitive. Testing the effect of LuxR on *ainSR* is potentially complicated by the role of the Ain system in regulating LuxR, most notably via LitR^17^, but also potentially via C8-HSL affecting LuxR’s autoregulatory activity^32^. We eliminated such complicating feedback loops by utilizing a set of engineered strains lacking both AHL synthases and *litR* while also using a constitutive non-native promoter to drive *luxR* transcription^21^. Using these engineered strains, we found that addition of 3OC6-HSL significantly decreased activity of a P_*ainS*_ *-gfp* reporter (*P* < 0.05) only when *luxR* was present (Fig. 3A). This effect of 3OC6-HSL appeared to be dose-dependent over a physiologically relevant range from 10 to 100 nM, although this effect was more evident with *luxR* from MJ1 than *luxR* from ES114 (Fig. 3B). At high concentrations C8-HSL can inhibit activation of the *lux* operon by 3OC6-HSL-LuxR^15,16,21^, and we similarly found that C8-HSL could significantly (*P* < 0.05) alleviate the repressive effect of 3OC6-HSL-LuxR on *ainS* reporter activity (Fig. 3A). Addition of 500 nM C8-HSL alone had no apparent effect (*P* > 0.05) on the reporter (Fig. 3A).

**Figure 4:**
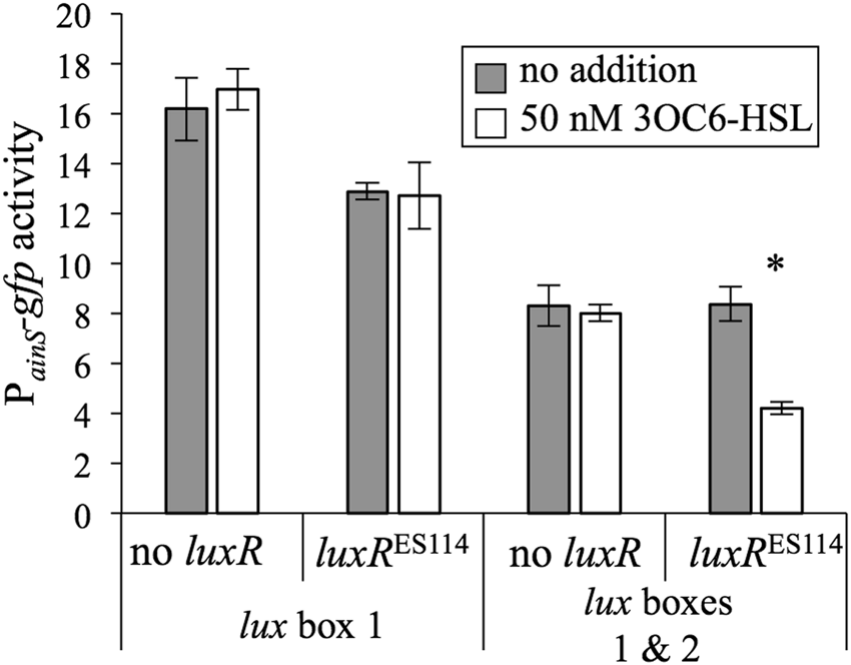
“*lux box* 2” is required for repression of *ainSR* promoter-reporter by LuxR-3OC6-HSL. Fluorescence of cells harboring P_*ainS*_ *-gfp* reporter pHK12 grown in SWTO to OD_595_ ~2.5. Strains with *luxR* ^ES114^ or no *luxR* harboring P_*ainS*_ *-gfp* reporter plasmids pHK156 (with *lux* box 1) or pHK12 (both *lux* boxes) were grown with 50 nM 3OC6-HSL (white bars) or no addition (grey bars). Strains harboring these reporters lack AHL synthases (∆*luxI* ∆*ainS*) and feedback regulation of *luxR* (*litR*::*ermR* P_con_ *-luxR*). Bars indicate standard error (*n* = 3), and asterisk indicates a significant difference in reporter activity upon addition of 50 nM 3OC6-HSL (*P* < 0.05).

As noted above, a previous study^45^ highlighted a potential *lux* box upstream of *ainS* (“*lux* box 1” - Fig. 2D), and we found a second putative *lux* box (“*lux* box 2” - Fig. 2D). The plasmid-based P_*ainS*_-*gfp* reporter described in the experiments above (pHK12) includes both of these *lux* boxes. We generated a second reporter plasmid (pHK156) containing only *lux* box 1 and TSS1, without *lux* box 2 or TSS2 (Fig. 2D), and a comparison of the reporters indicated that repression by 3OC6-HSL-LuxR was lost when only *lux* box 1 and TSS1 were included (Fig. 4). As discussed below, these data suggest the importance of the downstream *lux* box and promoter for LuxR-mediated regulation of *ainS*.

**Figure 5:**
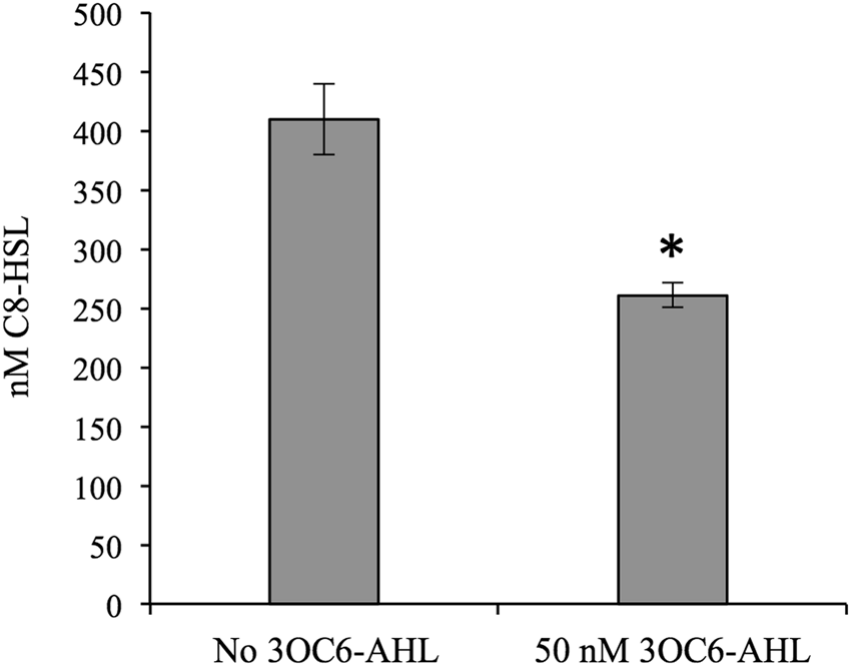
C8-HSL accumulation by strain JHK091 is affected by 3OC6-HSL (-AHL). The asterisk indicates a significant difference between 0 and 50 nM 3OC6-HSL (*P* < 0.05). Error bars indicate standard error (*n* = 2).

We also tested the effect of LuxR-3OC6-HSL on the accumulation of C8-HSL, which is the product of AinS. We used strain JHK091 where *luxR* is again disconnected from native *ain*-influenced regulation using a *litR*::*ermR* mutation and a constitutive non-native *luxR* promoter, but in this case *ainS* was present. When JHK091 was grown with added 3OC6-HSL, it produced significantly less C8-HSL (Fig. 5). Thus, our data show that LuxR can both repress transcription from the *ainS* promoter (Figs 3 and 4) and diminish output of C8-HSL (Fig. 5).

### Correlation between IR1 and C8-HSL accumulation

**Figure 6:**
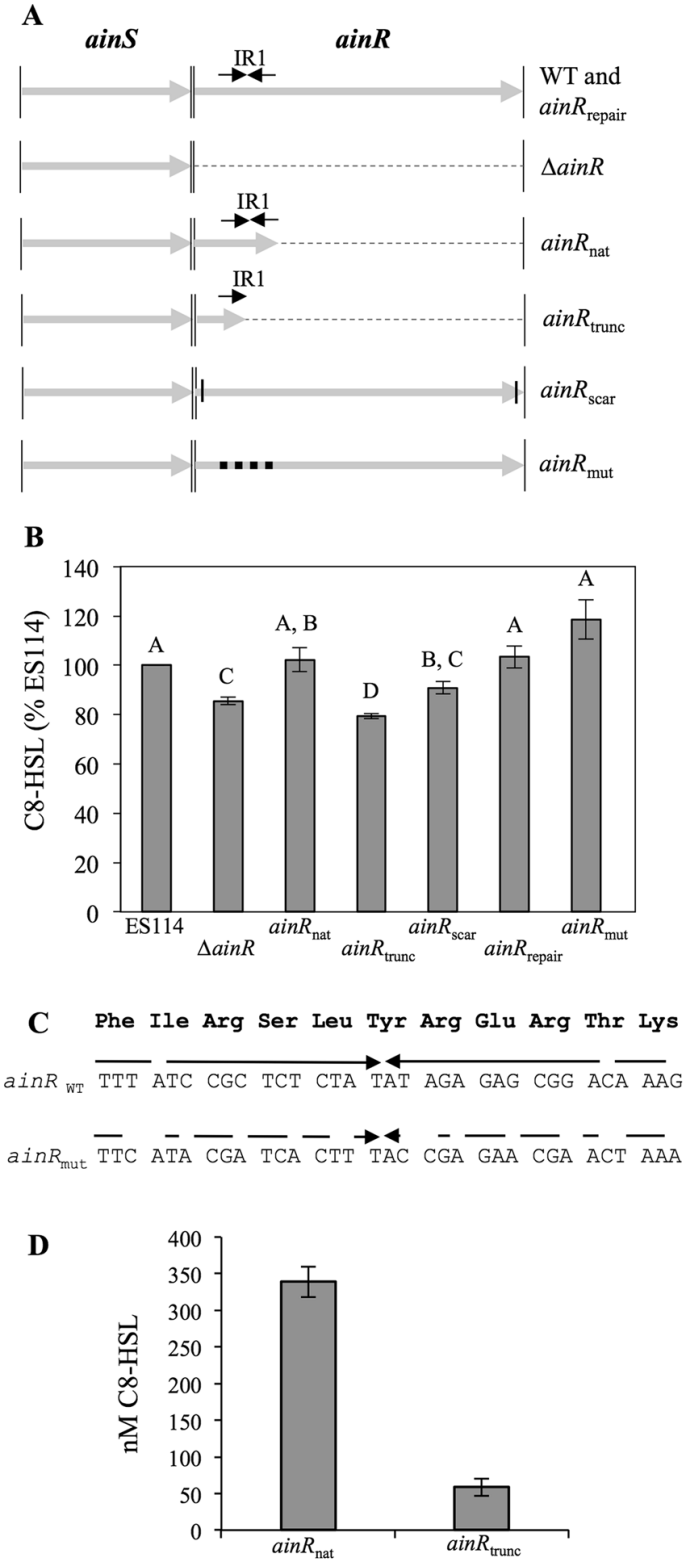
Effects of *ainR* sequence on C8-HSL accumulation. Panel A: Illustration of *ainR* alleles in this study. The *ainS* and *ainR* genes are shown as grey arrows delineated by thin vertical lines. Dashed lines correspond to deletions in *ainR*. Each repeat in IR1 is shown as a black arrow, and in *ainR*_mut_ the IR is altered to scramble the inverted repeat without changing the amino acid sequence. In *ainR*_scar_, short horizontal lines near the *ainR* termini indicate 6-bp insertions from restriction enzyme sites. Panel B: C8-HSL accumulation in cultures of strains ES114, JHK003 (∆*ainR*), JHK055 (*ainR*_trunc_), JHK056 (*ainR*_nat_), JHK115 (*ainR*_scar_), JHK119 (*ainR*_repair_), JHK120 (*ainR*_mut_) grown with shaking in SWTO medium to an OD_595_ ~1.5. Letters indicate significant differences (*P* < 0.05) in ANOVA test. Panel C: Alignment of IR1 region in wild type (*ainRWT*)and the targeted mutant *ainR*_mut_ showing conservation of amino acids encoded and increased number of mismatches in the inverted repeat, which are depicted as gaps in arrows. Panel D: C8-HSL accumulation in cultures of *E*. *coli* MG1655 carrying the *ainR*_nat_ and *ainR*_trunc_ alleles on pHK103 and pHK102, respectively, grown shaking in LB to OD_595_ ~1.5. Bars indicate standard error (*n* = 2).

The discovery of IR1 (Fig. 2C) was intriguing given the previous evidence that having *ainR* present *in cis* with *ainS* somehow affected C8-HSL output^13^. We therefore hypothesized that IR1 in *ainR* might have a post-transcriptional effect on AinS expression, thus accounting for the decreased C8-HSL production of a ∆*ainR* mutant. To test the correlation between IR1 and C8-HSL, we constructed *ainR* variants truncated immediately after IR1 (*ainR*_nat_) or in IR1 (*ainR*_trunc_) (Fig. 6A). We found that truncation in IR1 resulted in decreased C8-HSL accumulation, as did full deletion of *ainR*; however, truncation of *ainR* after IR1 yielded wild-type levels of C8-HSL (Fig. 6B).

The above experiments with ES114 and the ∆*ainR*, *ainR*_nat_, and *ainR*_trunc_ mutants showed a correlation between the presence or absence of the full IR1 and higher or lower C8-HSL, respectively; however, three additional experiments suggested a more complex, context-dependent role of IR1. First, we reintroduced full-length *ainR* into the ∆*ainR* mutant by cloning *ainR* into an AvrII restriction site created within the ∆*ainR* allele. Although this process re-introduced the complete IR1, it also introduced a 6-bp restriction site scar immediately after the *ainR* start codon, generating a new allele that we designated *ainR*_scar_ (Fig. 6A). Re-introducing IR1 on the *ainR*_scar_ allele did not restore wild-type C8-HSL levels (Fig. 6B). To test whether a second-site mutation in the *ainR*_scar_ mutant was responsible for its unexpected phenotype, we crossed the wild-type *ainR* sequence into the *ainR*_scar_ mutant (*ainR*_repair_ – Fig. 6A), which restored C8-HSL to wild-type levels. We also created an *ainR*_mut_ allele (Fig. 6A), in which IR1’s DNA symmetry was disrupted while preserving the amino acid sequence of AinR (Fig. 6C). Despite lacking IR1, the strain with *ainR*_mut_ produced wild-type levels of C8-HSL (Fig. 6B). Taken together, it appears that IR1 *in cis* can influence C8-HSL output, but this effect is influenced by the context of sequences both in and outside of IR1.

We hypothesized that IR1 preserves *ainS* mRNA by inhibiting 3′ RNA exonuclease activity from reaching and degrading the *ainS* coding part of the *ainSR* transcript, and we hoped to test this by exploiting defined RNase mutants in *E*. *coli*. When we placed constructs expressing *ainS*-*ainR*_trunc_ and *ainS*-*ainR*_nat_ into *E*. *coli* MG1655, we observed a similar pattern of C8-HSL accumulation as we saw for these alleles in *V*. *fischeri*, with even more dramatic differences (Fig. 6D). However, when we screened C8-HSL output in transgenic *ainS*-containing *E*. *coli* mutants^51–53^ that lack RNases D (*rnd*), PH (*rph*), T (*rnt*), R (*rnr*), or Z (*rnz*), we still saw higher C8-HSL output from *ainS*-*ainR*_nat_ than from *ainS*-*ainR*_trunc_ (see Supplementary Table S1). Furthermore, qRT-PCR revealed indistinguishable levels of *ainS* transcript copy number in ES114 and all of the *ainR* mutant variants shown in Fig. 6A (see Supplementary Figure S2). Thus we found no evidence that IR1 influences *ainS* transcript stability.

## Discussion

*V*. *fischeri* uses the *luxIR* and *ainSR* AHL-based PS systems to control symbiotic phenotypes^1,3,54^, with the *ain* system being the first of these to be activated and priming the *luxIR* system through a signaling cascade conserved amongst members of the *Vibrionaceae* family. Despite the placement of *ainSR* atop this signaling cascade, only recently have we begun to understand the regulation of this system^2,13,24,55^. In this study, we expanded upon those findings by exploiting comparative genomic analyses and mapping the transcriptional start sites for *ainSR* (Fig. 2), which we confirmed are co-transcribed. Our results shed new light on regulation of the *ainSR* operon and its connection to *luxRI* in the PS circuitry of *V*. *fischeri* (see Supplementary Figure S3).

Previous studies showed that LitR and cAMP-CRP regulated *ainS* ^2,17,24^. A LitR binding site sequence has remained elusive, but binding studies confirmed cAMP-CRP interaction with a near-canonical recognition sequence upstream of *ainS* ^24^, at a site that is reasonably well conserved across strains (Fig. 2D). It was previously noted that CRP appeared to elicit both a LitR-dependent activation and LitR-independent repression of the *ainS* promoter, and the mapping of transcriptional start sites (Fig. 2D) allows further interpretation of these observations. The speculated “class III” CRP-dependent activation in conjunction with LitR^24^ could only realistically occur at the further downstream transcriptional start site, TSS2 (Fig. 2D). On the other hand, the known CRP binding site appears to overlap the −35 promoter element associated with TSS1 (Fig. 2D), and this might lead to repression. Further study is warranted to make firm conclusions about regulatory mechanisms, and defining LitR-DNA interactions would be especially useful in this regard.

Hierarchical activation of PS systems, such as the jump-starting of LuxIR by AinSR, is a common feature of bacteria possessing multiple PS systems^56,57^. We have now shown a negative feedback loop also exists between the second system and the first in *V*. *fischeri* ES114. LuxR represses the *ainS* promoter, and this activity responds to 3OC6- and C8-HSL in much the same way that these pheromones activate (3OC6-HSL) or antagonize (C8-HSL in the presence of 3OC6-HSL) LuxR’s stimulation of transcription at the *luxI* promoter (Fig. 3A). During colonization of the host, LuxR repression of *ainS* might lead to lower concentrations of C8-HSL, while *luxIR* expression increases during establishment and progression of the symbiosis.

Negative feedback loops are known in other bacterial cell-cell signaling systems. In *Pseudomonas aeruginosa*, RsaL serves a negative regulator of the *lasIR* PS system, binding its activator LasR and preventing it from activating expression of the *lasI* AHL synthase, thus maintaining signal levels during growth^58,59^. In a similar but more complicated scheme, *Sinorhizobium meliloti* ExpR activates expression of the AHL-synthase *sinI* and represses expression of a second *sinI* activator, *sinR* ^60^, thus, like RsaL, ultimately repressing the expression of its cognate-signal synthase. Perhaps a closer parallel to *V*. *fischeri* is found in *Burkholderia cenocepacia*, which contains CepI/CepR and CciI/CciR AHL-based PS systems. In *B*. *cenocepacia* CepR is required for *cciIR* induction, but CciR represses *cepI* ^61^, forming an inter-system negative feedback loop. As more bacteria with multiple PS systems are investigated, such inter-circuit feedback loops may become more apparent.

Identification of this negative feedback regulation by 3OC6-HSL-LuxR on *ainSR* is somewhat surprising considering that despite such regulation being postulated upon the discovery of these genes^45^, *ainSR* was not identified as a target gene in multiple studies of LuxR-dependent regulation^49,50,62^. In this regard it is worth noting that the effect of LuxR repression is small (Figs 3 and 4) and might be further obscured by feedback loops that we have deliberately removed from our experimental setup. An early proteomic analysis of the LuxR regulon therefore might easily have missed a small change in AinS levels^50^. In a setup closer to ours, Qin and colleagues saw no effect of LuxR on a P_*ainS*_-*lacZ* transcriptional reporter, but used sequences of *V*. *fischeri* MJ1 in transgenic *E*. *coli*, which may account for differences between our results in *V*. *fischeri* ES114^62^.

Perhaps the hardest data to reconcile with our own are that of Antunes *et al*.^49^, who used a microarray to assess the effect of adding 3OC6-HSL on the ES114 LuxR regulon. That study differed from ours in that it was performed in a background of endogenous 3OC6-HSL and C8-HSL, and perhaps more importantly in that it also included LitR-dependent positive feedback, which as noted above might obscure the negative feedback we observed. One might then question whether or not LuxR-mediated repression of *ainSR* is relevant in wild type, if its detection requires decoupling from LitR-mediated feedback. While this poses a reasonable question, it seems unlikely that LuxR-mediated repression of *ainSR* would represent a coincidental artifact, with a LuxR binding site overlapping an *ainSR* promoter occurring by chance. We speculate that this regulation evolved due to a fitness advantage conferred in some situation(s), for example during host colonization, where conditions undoubtedly differ from any of these experimental setups in batch broth cultures. For example, host-mediated C8-HSL turnover could dramatically affect regulation during the hierarchical activation of the two AHL-based systems. Further studies of this regulatory cascade during a model symbiotic infection will help resolve these issues.

One of the more striking findings of our analysis of the *ainSR* locus was the presence of a small region of relatively high conservation between ES114 and MJ11, and to a lesser degree conserved in *V*. *salmonicida*, including the inverted repeat elements IR1 and IR2 within the 5′ part of *ainR* (Fig. 2A and C). The sequences of AinS and AinR have diverged more than other protein components of the core *Vibrio* PS system (i.e. LuxU, LuxO, LitR)^34^, but IR1 in particular was striking and almost completely conserved.

We suspected that these IR sequences in *ainR* related to previously unexplained phenotypes of ∆*ainR* mutants. Ray and Visick reported a luminescence defect in a ∆*ainR* mutant, which is the opposite of the predicted effect based on our understanding of the regulatory system and its orthologs in *V*. *harveyi* ^18^. We also subsequently showed less C8-HSL accumulation in ∆*ainR* strains, independent of the positive feedback in the Ain system mediated by LitR^13^, which again was unexpected and if anything contrary to our understanding of the PS circuitry. Importantly, we found *ainR* must be present *in cis* with *ainS* to alleviate this defect, suggesting that the effects were related to the linkage between *ainS* and *ainR*, rather than AinR^13^. We have now confirmed that *ainS* and *ainR* are cotranscribed, and we hypothesized that IR1 in *ainR* protected the *ainS* portion of the transcript from degredation, resulting in more AinS, C8-HSL, and luminescence in wild type than in the ∆*ainR* mutant. However, although truncations in *ainR* supported this idea, other *ainR* alleles suggested a more complex regulatory mechanism, as there was not a consistent clear-cut difference between the presence or absence of IR1 (Fig. 6). We also saw no difference in *ainS* mRNA levels in wild type and the ∆*ainR* mutant (data not shown). These results indicate that the mechanism by which *ainR* sequence influences *ainS* and C8-HSL production is not the simple transcript-stability model that we initially proposed. Further examination of AinS protein levels using these IR1 mutants may reveal clues to how *ainR* sequence affects C8-HSL production. Whatever the mechanism, post-transcriptional regulation may allow *ainSR* to be co-transcribed while AinS and AinR are expressed in different stoichiometries.

Sequence comparisons at the *luxIR* locus revealed conserved regulatory elements^34^, and here a similar bioinformatic approach helped identify potential regulatory mechanisms in the *ainSR* PS system. As more genome sequences for different *V*. *fischeri* strains become available, this comparative approach may become even more useful in identifying regulatory elements at these and other loci. In this study, new puzzling questions have been revealed and remain unanswered, including; (i) what is the actual translational start(s) of AinS, considering previous annotations are inconsistent with transcripts arising at TSS2, (ii) what is the mechanistic role of IR1 in *ainR*, and (iii) is the negative feedback loop between LuxR and *ainSR* relevant to PS during symbiotic infection. Further investigation should also help clarify LitR’s relation to *ainSR* regulation (e.g. its binding site) and help determine whether any other factors control this locus.

## Materials and Methods

### Bacteria, growth media, and reagents

Bacterial strains are listed and briefly described in Table 1. *V*. *fischeri* ES114 was the wild-type strain used throughout^36^. Plasmids were transformed into *Escherichia coli* strain DH5α^63^ or DH5αλ*pir* ^64^ in the case of plasmids with the R6K origin of replication. *E*. *coli* strain MG1655^65^ and its derivatives were used as recipients for plasmids expressing different *ainR* alleles as described below. *E*. *coli* was grown in LB medium^66^ or brain heart infusion (BHI) medium, and *V*. *fischeri* was grown in LBS^67^ or SWTO^27^. Solid media were prepared with 15 g L^−1^ agar. For selection of *E*. *coli*, chloramphenicol (Cam) and kanamycin (Kan) were added to LB at final concentrations of 20 and 100 μg ml^−1^, respectively, and erythromycin (Erm) was added to BHI at a final concentration of 150 μg ml^−1^. For selection of *V*. *fischeri* on LBS, the concentrations of Cam, Erm, and Kan used were 2, 5, and 100 μg ml^−1^, respectively. 3OC6-HSL and C8-HSL were obtained from Sigma-Aldrich (St. Louis, MO).

**Table 1:** Bacterial strains and plasmids used in this study.

Strain, plasmid, or oligonucleotide	Relevant characteristics^a^	Source or reference
Strains		
***E. coli***		
CC118λpir	Δ(*ara-leu*) *araD Δlac74 galE galK phoA20 thi-1 rpsE rpsB argE*(Am) *recA* λ*pir*	68
DH5α	F-φ80d*lacZ*ΔM15 ∆(*lacZYA-argF*)U169 *deoR supE44 hsdR17 recA1 endA1 gyrA96 thi-1 relA1*	63
DH5α λpir	DH5α lysogenized with λ*pir*	64
JW1644-5	∆(*araD-araB*)567 ∆(*lacZ4787*(::*rrnB-3*)) λ^-^ ∆(*rnt-730*::*kanR*) *rph-1* F^-^ ∆(*rhaD-rhaB*)*568 hsd514*	52
JW1793-1	∆(*araD-araB*)567 ∆(*lacZ4787*(::*rrnB-3*)) λ^-^ ∆(*rnd-729*::*kanR*) *rph-1* F^-^ ∆(*rhaD-rhaB*)*568 hsd514*	51
JW3618-2	∆(*araD-araB*)567 ∆(*lacZ4787*(::*rrnB-3*)) λ^-^ ∆(*rph-749*::*kanR*) *rph-1* F^-^ ∆(*rhaD-rhaB*)*568 hsd514*	51
JW5741-1	∆(*araD-araB*)567 ∆(*lacZ4787*(::*rrnB-3*)) λ^-^ ∆(*rnr-729*::*kanR*) *rph-1* F^-^ ∆(*rhaD-rhaB*)*568 hsd514*	52
MG1655	F^–^ λ^–^ *ilvG* ^–^ *rfb-50 rph-1*	65
SK2595	*araD139 galE15 galK16* Δ(*ara-leu*)*7697 spoT1* λ^-^ *hsdR2* Δ(*codB-lacI*)*3 mcrA0 relA1 rpsL150 mcrB9999* ∆(*rnz-500*::*kanR*) ∆(*elaC500*::*kanR*)	53
*V*. *fischeri*		
DC22	C8-HSL bioreporter: ES114 ∆*ainS* ∆*luxR-luxI*, mutant *luxR* (MJ1-T33A, R67M, S116A, M135I), P_*luxI*_-*luxCDABEG*	24
DC43	ES114 Δ*ainS* Δ*luxI* P_con_-*luxR*_MJ1_ P_*luxI*_-*luxCDABEG*	21
DJ01	ES114 Δ*ainS* Δ*luxI* P_con_-*luxR*_ES114_ P_*luxI*_-*luxCDABEG*	21
ES114	Wild-type isolate from *E*. *scolopes*	36
JB18	ES114 *litR*::*ermR*	13
JHK003	ES114 ∆*ainR*	13
JHK007	ES114 Δ*ainS* Δ*luxIR* P_*luxI*_-*luxCDABEG*	13
JHK045	ES114 Δ*ainS* Δ*luxI* P_con_-*luxR* ^ES114^ P_*luxI*_-*luxCDABEG litR*::*ermR*	This study
JHK046	ES114 Δ*ainS* Δ*luxIR* P_*luxI*_-*luxCDABEG litR*::*ermR*	This study
JHK055	ES114 *ainR*_trunc_ (Δ30-815)	This study
JHK056	ES114 *ainR*_nat_ (Δ36-815)	This study
JHK091	ES114 Δ*luxI* P_con_-*luxR* ^MJ1^ P_*luxI*_-*luxCDABEG litR*::*ermR*	This study
JHK099	ES114 Δ*ainS* Δ*luxI* P_con_-*luxR* ^MJ1^ P_*luxI*_-*luxCDABEG litR*::*ermR*	This study
JHK114	ES114 Δ*ainR*	This study
JHK115	ES114 *ainR*_scar_	This study
JHK119	ES114 *ainR*_scar*-*repaired_	This study
JHK120	ES114 *ainR*_mut_	This study
NL60	ES114 ∆*ainS*	23
NL63	ES114 ∆*ainS luxI*	13
**Plasmids** ^**b**^		
pCR-Blunt	PCR product cloning vector; ColE1 *oriV kanR*	Invitrogen
pCR-Blunt-II-TOPO	PCR product cloning vector; ColE1 *oriV kanR*	Invitrogen
pDJ01	P_con_-*luxR*_ES114_ P_*luxI*_-*luxCDABEG ColE1*, R6K*γ*, *oriT*_RP4_, *kanR*, *camR*	21
pEVS104	Conjugative helper plasmid; R6Kγ *oriT*_RP4_ *kanR*	69
pEVS118	Suicide vector; R6Kγ, *oriT*_RP4_, *camR*	64
pEVS122	Suicide vector; R6Kγ, *oriT*_RP4_, *ermR*, *lacZ*α	64
pHK12	P_*ainS*_-*gfp* P_*con*_ *-mCherry* in pJLS27; pES213, R6Kγ *oriT*_RP4_ *kanR*, *camR*	70
pHK34	*ainR*_trunc_ (Δ30-815) in pCR-Blunt; ColE1 *oriV kanR*	This study
pHK37	*ainR*_trunc_ (Δ30-815) allele; ColE1 R6Kγ *oriV oriT*_RP4_ *camR kanR*	This study
pHK75	1500-bp *ainR* downstream in pEVS122; R6Kγ *oriT*_RP4_ *ermR lacZ*α	This study
pHK76	*ainR*_nat_ (Δ36-815) allele; R6Kγ *oriT*_RP4_ *ermR lacZ*α	This study
pHK93	P_*ainS*_-*ainR*_trunc_ (Δ36-815) in pCR-Blunt; ColE1 *oriV kanR*	This study
pHK94	P_*ainS*_-*ainR*_nat_ (Δ30-815) in pCR-Blunt; ColE1 *oriV kanR*	This study
pHK95	P_*ainS*_-*ainR* in pCR-Blunt; ColE1 *oriV kanR*	This study
pHK102	P_*ainS*_-*ainR*_trunc_ (Δ30-815) allele; ColE1 R6Kγ *oriV oriT*_RP4_ *kanR camR*	This study
pHK103	P_*ainS*_-*ainR*_nat_ (Δ36-815) allele; ColE1 R6Kγ *oriV oriT*_RP4_ *kanR camR*	This study
pHK104	P_*ainS*_-*ainR* allele; ColE1 R6Kγ *oriV oriT*_RP4_ *kanR camR*	This study
pHK129	*ainSR* ClaI-NdeI fragment with mutated IR1 (*ainR_*IR_conAA) in pCR-Blunt; ColE1 *oriV kanR*	This study
pHK135	1,350-bp upstream of *ainR* in pCR-Blunt; ColE1 *oriV kanR*	This study
pHK136	∆*ainR*, 1,480-bp downstream *ainR* in pHK135; ColE1 *oriV kanR*	This study
pHK137	*ainR*_scar_ in pHK136; ColE1 *oriV kanR*	This study
pHK138	∆*ainR* allele; ColE1 R6Kγ *oriT*_RP4_ *kanR camR*	This study
pHK139	*ainR*_scar_ allele; ColE1 R6Kγ *oriT*_RP4_ *kanR camR*	This study
pHK152	*ainR*_mut_ in pHK95; ColE1 *oriV kanR*	This study
pHK153	*ainR*_mut_ allele; ColE1 R6Kγ *oriT*_RP4_ *kanR camR*	This study
pHK156	P_*ainS*_-*gfp* (truncated) P_*con*_ *-mCherry* in pJLS27; pES213, R6Kγ *oriT*_RP4_ *kanR*, *camR*	This study
pJLS27	Promoterless *gfp*, P_*con*_ *-mCherry* pES213, R6Kγ *oriT*_RP4_ *kanR*	26
pJLB95	*litR*::*ermR* (opposite) allele; ColE1 *camR ermR*	13
**Oligonucleotides** ^**c**^		
pr_HK01	GGATCTGGCTTTTAAAAAATGCATCATCTGC	This study
pr_HK02	CATCTAGATGACGATGAAGTACAGATATTGGTTTATGAAT	This study
pr_HK03	GGGGCATGCAGAACCAAGACCTGCTCGTGCTAA	70
pr_HK13	AGCGCCCAATACGCAAACC	This study
pr_HK14	CCGGCGTGTCAATAATATCACTCTGTACA	This study
pr_HK17.2	CATGGGATCCTAGAGAGCGGATAAAATACCCTACCCAA	This study
pr_HK27.3	CATGGGTACCAGAACCAAGACCTGCTCGTGCTAA	This study
pr_HK28.2	CATGGGATCCTAAGGGTTTACCTTTGTCCGCTCTCTA	This study
pr_HK40.1	CATGGGATCCATAAGTGGTTATAACACCGATAAAAAAATAGCC	This study
pr_HK41.4	CATGGCATGCTGAAGGTGCTTGCTATTACTGATCA	This study
pr_HK126	CATGGGATCCTGAAGGTGCTTGCTATTACTGATC	This study
pr_HK136	CATGGCTAGCTTAACTACTTTACCTAAAGTTTATTTAC	This study
pr_HK137	CATGGCTAGCTAACCACTTATCTACGACCT	This study
pr_HK144	AAAATAAGTATTCCAAATTTCCAA	This study
pr_HK146	AAAGTACTCATAACACCACTACC	This study
pr_NL28.3	GGGCCTAGGCATTTATATAAAACTCACTGA TTTCGAAGTTT	23
pr_NL29	GGGGCCTAGGTAACACCGATAAAAAAATAGCCAGAAC	23
pr_NL35	GAGTCCGTTAGCAAGGTCACACTTTGTTG	23
pr_NL63	GGGCCTAGGCTACTCTTTTATAAATTCATATTGCAGGTTTT	23
pr_NL89	AAATCTAAGGGTTTACCTTTGTCCGCTCTC	24
pr_NL108	GGCGGAACGATTGGAAATTTGGAATACTTATTTTCAACATC	24
pr_NL109	CAGTACTGCATTTCAAAAGACAACCAAAAACTTTGATAGCC	24
QO	CCAGTGAGCAGAGTGACG	72
QI	GAGGACTCGAGCTCAAGC	72
QT	CCAGTGAGCAGAGTGACGAGGACTCGAGCTCAAGCTTTTTTTTTTTTTTTTT	72

^a^Drug resistance abbreviations used: *camR*, chloramphenicol resistance; *ermR*, erythromycin resistance; and *kanR*, kanamycin resistance (*aph*).

^*b*^All alleles cloned in this study are from *V*. *fischeri* strain ES114. Replication origin(s) of each vector are listed as R6Kγ, ColE1, *oriV* and/or pES213. Plasmids based on pES213 are stable and do not require antibiotic selection for maintenance.

^c^All oligonucleotides are shown 5′ to 3′. Restriction enzyme recognition sequences are underlined.

### Molecular genetic techniques

Oligonucleotides and plasmids are listed in Table 1 and the latter were constructed using standard techniques, with enzymes and other materials described previously^13^. To generate the P_*ainS*_-*gfp* reporter pHK156, a 428-bp fragment extending upstream of *ainS* was PCR amplified with primers pr_HK03 and pr_NL63. The resulting amplicon was digested with SphI and AvrII and ligated into SphI- and XbaI-digested pJLS27.

To generate the *∆ainR* deletion allele on allelic exchange vector pHK138, a 1,350-bp fragment upstream of *ainR* including the start codon was PCR amplified using primers pr_HK146 and pr_NL28.3 and cloned into pCR-Blunt to generate pHK135. The 1,519-bp region downstream of *ainR* including the stop codon was PCR amplified using primers pr_NL29 and pr_HK126, digested with BamH1 and AvrII, and ligated into BamHI- and AvrII-digested pHK135 to create pHK136. This plasmid contains a unique AvrII restriction site between the *ainR* start and stop codons along with sequences flanking *ainR*. To reintroduce *ainR* into *∆ainR* mutants, wild-type *ainR* was PCR amplified using primers pr_HK136 and pr_HK137 and this fragment was digested with NheI and ligated into AvrII-digested pHK136 to generate pHK137. As a result of these cloning steps, *ainR* on pHK137 differs from wild type in that it contains two 6-bp insertions, one immediately following the start codon and another preceding the stop codon, and we refer to the corresponding allele as *ainR*_scar_. Mobilizable ∆*ainR* and *ainR*_scar_ allelic exchange vectors were generated by digesting pHK136 and pHK137 with BamHI and ligating them to BamHI-digested pEVS118^64^, which contains a conjugative origin of transfer, generating pHK138 and pHK139, respectively.

To generate the *ainR*_trunc_ allele on an allelic exchange vector, in which *ainR* is truncated after the first 16-bp of the inverted repeat IR1, 428 bp upstream of *ainS* through the first half of the *ainR* inverted repeat (see Results) was PCR amplified using primers pr_HK17.2 and pr_HK28.2. A 1,500-bp region comprising the final 15 bp of *ainR* and sequence downstream of *ainR* was PCR-amplified using primers pr_HK40.1 and pr_HK41.4. The two amplicons were digested with BamHI, ligated together, and the combined fragment was blunt-end cloned into pCR-Blunt-II-TOPO to generate pHK34. This plasmid was then digested with KpnI and ligated to KpnI-digested pEVS118 to generate pHK37. To generate the *ainR*_nat_ allele on allelic exchange vector pHK76, in which *ainR* is truncated after IR1, 1,500-bp downstream of *ainR* was again amplified using primer pair pr_HK40.1 and pr_HK41.4. This amplicon was digested with BamHI and SphI and ligated with BamHI and SphI-digested pEVS122^64^ to generate pHK75. The fragment from 428-bp upstream of *ainS* through IR1 was PCR amplified with primers pr_HK27.3 and pr_HK28.2. The resulting amplicon was digested with BamHI and KpnI and ligated into similarly digested pHK75 to generate the *ainR*_nat_ allele on pHK76.

To place *ainS* and variants of *ainR* in *E*. *coli* on isogenic plasmid constructs, the fragment containing the 428-bp region upstream of *ainS* through 1,500-bp downstream of the *ainR* stop codon in strains ES114, JHK055, and JHK056 (described below) were PCR-amplified using primers pr_HK27.3 and pr_HK41.4 and cloned into pCR-Blunt-II-TOPO in the same orientation generating pHK95, pHK93 and pHK94, respectively. To add a selectable marker compatible with kanamycin-resistant *E*. *coli* RNase mutants, each plasmid was then digested using KpnI and SpeI and ligated with similarly digested pEVS118, which encodes resistance to Cam, to generate pHK104, pHK102 and pHK103, respectively.

To assess the function of an inverted repeat (IR1) in *ainR*, a synthetic DNA fragment (Integrated DNA Technologies, Coralville, IA) was designed to preserve the amino acid sequence of AinR while also disrupting the mirror symmetry of IR1 in *ainR*. This sequence, *ainR*_IR_conAA, is contained in an 844-bp ClaI to NdeI fragment. The synthetic fragment was cloned into pCR-Blunt to generate pHK129 and then PCR-amplified using primers pr_HK13 and pr_HK14. The resulting amplicon was ClaI- and NdeI-digested and ligated into similarly digested pHK95 to replace the native *ainR* sequence and generate pHK152. This plasmid was then digested with KpnI and SpeI and ligated with similarly digested pEVS118 to generate the mobilizable allelic exchange vector pHK153.

Mutant alleles were transferred from *E*. *coli* into *V*. *fischeri* on plasmids by triparental matings using the conjugative helper strain CC118λ*pir* pEVS104^68,69^. Recombination and marker exchange were identified by screening for antibiotic resistance, and putative mutants were tested by PCR. In this way, the allele on pJLB95^13^ was introduced into DC43, DJ01, and JHK007 to generate JHK099, JHK045, and JHK046, respectively. To generate strains with different *ainR* variants, the alleles on pHK36, pHK76, pHK138, or pHK153 were introduced into ES114 to generate strains JHK055, JHK056, JHK114, and JHK120, respectively. JHK114 was subsequently used as the parent strain for the reintroduction of the *ainR*_scar_ allele on pHK139, thus generating JHK115. The *ainR*_scar_ allele in JHK115 was then recombinationally repaired using the native *ainR* locus on pHK104, generating strain JHK119. The P_con_-*luxR* P_*luxI*_-*luxCDABEG ∆luxI* locus on pDJ01 was introduced into the *litR*::*ermR* strain JB18^13^ to generate strain JHK091.

### Luminescence measurements

Overnight *V*. *fischeri* cultures were diluted 1:1,000 in 25 ml SWTO in 125-ml flasks and incubated with shaking (200 rpm) at 24 °C. At regular intervals, the optical density at 595 nm (OD_595_) was measured for 500-μl samples using a BioPhotometer (Brinkman Instruments, Westbury, NY). Relative luminescence was measured with a TD-20/20 luminomenter (Turner Designs, Sunnyvale, CA) immediately following shaking to aerate the sample. Specific luminescence was calculated as the luminescence per OD_595_.

### Transcriptional reporter assays

Strains harboring the P_*ainS*_ *-gfp* reporter plasmid pHK12^70^ or the promoterless parent vector pJLS27^26^ were grown overnight in LBS and subcultured 1:1,000 into 125-ml flasks containing 25 ml SWTO, with or without 3OC6-HSL or C8-HSL, and incubated with shaking (200 rpm) at 24 °C. At regular intervals, 200-µl samples were aliquoted into clear-bottomed, black-walled, 96-well plates, where green fluorescence and OD_595_ were measured using a Synergy 2 plate reader (BioTek, Winooski, VT). Fluorescence values are reported from cultures at a similar cell density (OD_595_), as indicated.

### C8-HSL bioassays

C8-HSL accumulation was assessed as previously described^24^. Briefly, culture supernatants were extracted with acidified ethyl acetate, extracts were dried and resuspended in SWTO, and C8-HSL levels were determined by comparison to standards using the bioassay strain DC22.

### Characterization of ain transcript

Overnight cultures were diluted 1:1,000 in SWTO and grown to an OD_595_ ~0.5, at which point total RNA was extracted using the RNASnap method of Stead *et al*.^71^, followed by precipitation with sodium acetate and ethanol. RQ1 DNase (Promega, Madison, WI) was used to remove genomic DNA from samples according to the manufacturer’s protocol. 50 ng of DNA-free RNA was used as template for reverse transcription using the Superscript III First Strand cDNA synthesis kit (Invitrogen, Orange, CA) with random hexamers according to the manufacturer’s protocol. The resulting cDNA was diluted 1/10 in a PCR reaction using the primers pr_HK01 and pr_NL89^24^, which encompass a 236-bp fragment spanning the junction of *ainS* and *ainR*. The resulting amplicon from ES114 cDNA was cloned and sequenced to confirm its identity.

The *ainS* transcriptional start site was determined by the rapid amplification of cDNA ends (RACE) method of Scotto-Lavino *et al*.^72^. DNA-free RNA was prepared from ES114 grown in SWTO medium at 24 °C to an OD_595_ ~0.5 as described above. One microgram of RNA was used as a template for cDNA generation using the SuperScript III First-Strand Synthesis system (Invitrogen) and the *ainS-*specific primer pr_NL35^23^ followed by RNA removal using RNase H (Invitrogen). Poly-A tails were then added to cDNA products using 250 ng cDNA and terminal transferase (New England Biolabs, Ipswich, MA). Tailed cDNAs were then diluted 1:25 and used as template for the first of two nested-PCR reactions using three primers^72^ QT, QO, and the *ainS-*specific primer pr_HK02. The PCR products were then cleaned and diluted 1:250 and used as template for a second nested-PCR reaction with primers QI and pr_HK144 followed by cloning into pCR-Blunt and sequencing to determine the origin of the mRNA.

### Quantitative RT-PCR analysis of ainS transcript

DNA-free RNA was prepared as it was for RACE analysis. 100 ng RNA was used as template for reverse transcription using either the SuperScript VILO (Invitrogen) or iScript (Bio-Rad, Hercules, CA) kits, according to the manufacturer’s protocol. Ten ng of RNase-treated cDNA was used as a template for qPCR using *ainS-*specific primers pr_NL108 and pr_NL109^24^ and the iQ SYBR-green qPCR supermix (Bio-Rad) using the MyiQ real-time PCR detection system. To generate standard curves, 10-fold serial dilutions of pHK95 were included during real-time analysis, and no-template and no-reverse transcriptase controls were included when appropriate.

### Data availability statement

The datasets generated and analyzed during the current study are available from the corresponding author upon reasonable request.

## Electronic supplementary material

Supplementary Information
