# Supplementary material for: Comparative analysis reveals regulatory motifs at the ainS/ainR pheromone-signaling locus of Vibrio fischeri
Source: Sci Rep. 2017 Sep 15;7:11734. doi: 10.1038/s41598-017-11967-7 (PMC5601948; doi:10.1038/s41598-017-11967-7)
Supplement: Supplementary file 1 — Supplementary Information [file 41598_2017_11967_MOESM1_ESM.pdf]

Supplementary information for:

Comparative analysis reveals regulatory motifs at the *ainS*/*ainR* pheromone-signaling locus of *Vibrio fischeri*

John H. Kimbrough and Eric V. Stabb

Department of Microbiology, University of Georgia, Athens, GA, USA

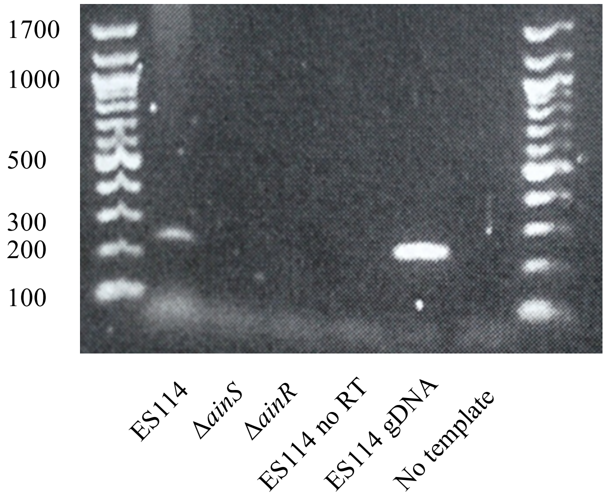

Supplemental Figure S1. *ainS* and *ainR* are co-transcribed. RNA was extracted from cultures of ES114, NL60 (Δ*ainS*), and JHK003 (Δ*ainR*). Following reverse transcription reactions, the resulting cDNA pools were examined by PCR with primers that amplify a 236-bp region spanning *ainS* and *ainR*. Sequencing confirmed the identity of the amplicon. Controls with no reverse transcriptase, ES114 genomic DNA, and no template are also shown. Molecular size standards, in base pairs, are shown on the left-hand side.

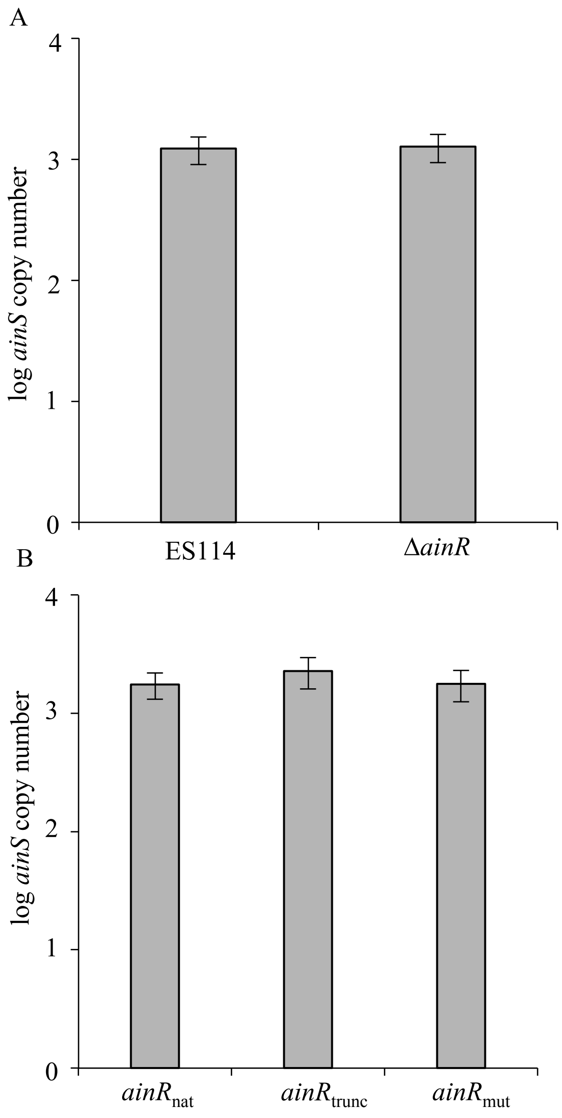

Supplemental Figure S2. *ainS* transcript number is not affected by mutations in *ainR* IR1. Quantititive RT-PCR determination of relative *ainS* mRNA copy number is reported for cDNA made from RNA that was isolated from cultures of A) ES114 (parent), JHK003 (Δ*ainR*) or B) JHK056 ($ainR_{nat}$), JHK055 ($ainR_{trunc}$), and JHK120 ($ainR_{mut}$). The cDNA was generated and assayed by qRT-PCR as described in *Materials and Methods.* RNA from NL60 (Δ*ainS*) served as a negative control and is not shown because it was below the limit of detection.

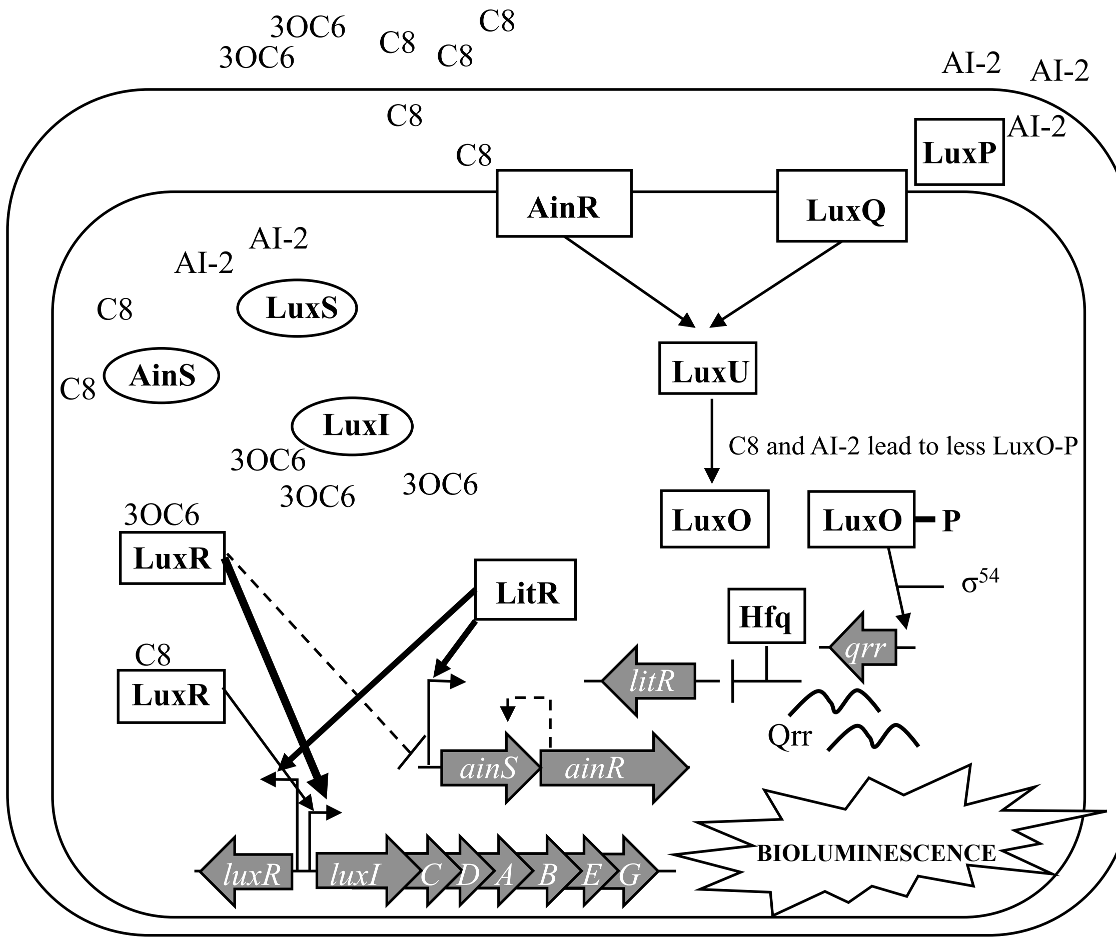

Supplemental Figure S3. Model of *V. fischeri* pheromone-signaling systems. The pheromone receptors AinR and LuxQ phosphorylate and de-phosphorylate LuxU, which phosphorylates LuxO, stimulating it to activate transcription of the sRNA *qrr*. Qrr represses expression of the "master regulator" LitR. As C8-HSL (indicated as C8) and AI-2 levels increase, the effects signals on AinR and LuxQ system shift to less LuxU-P, less LuxO-P, less Qrr, and more LitR. High C8-HSL and LitR levels increase luminescence through LuxR. LuxR is stimulated by both C8 and the product of LuxI (indicated as 3OC6). Dotted lines indicate the observed regulatory relationships described in this work. We propose that LuxR combined with 3OC6 represses *ainSR*, with this effect requiring "Lux box 2" (Fig 2) upstream of *ainSR*. We also propose that sequences with *ainR*, when *in cis* with *ainS*, positively affect output of the C8-HSL product of AinS through an unknown mechanism that appears correlated with a striking inverted repeat within *ainR*.

Supplemental Table S1. Fold difference in C8-HSL accumulation in *E. coli* RNase mutants expressing different *ainR* IR variants.

Strain[a]	Fold Difference[b]
MG1655	5.8
rnd	3.9
rph	3.1
rnt	6.5
rnr	3.2
rnz	3.6

[a]*E. coli* MG1655 and derived strains with mutations in *rnd* (JW1793-1), *rph* (JW3618-2), *rnt* (JW1644-5), *rnr* (JW5741-1), or *rnz* (SK2595) harboring either pHK103 ($ainS\text{-}ainR_{\mathrm{nat}}$) or pHK102 ($ainS\text{-}ainR_{\mathrm{trunc}}$) were grown shaking (200 r.p.m.) at 37°C in LB medium to an OD_{600} ~1.0 and C8-HSL was extracted and quantified as described in *Materials and Methods* ($n = 2$).

[b]Fold difference is the concentration of C8-HSL accumulated in cultures of a strain harboring the wild-type *ainSR* allele (pHK103) divided by C8-HSL accumulated in cultures of that same strain harboring this locus with *ainR* truncated such that IR1 is incomplete (pHK102). C8-HSL accumulation was not assessed for all strains in the same experiment, but each reported Fold Difference was calculated from C8-HSL levels assessed in parallel, in the same experiment, for cultures of that particular strain carrying pHK102 and pHK103.
